# Qualitative exploration of the lived experiences of loneliness in later life to inform technology development

**DOI:** 10.1080/17482631.2024.2398259

**Published:** 2024-09-20

**Authors:** Jessica Rees, Wei Liu, Jiana Canson, Lynda Crosby, Anthea Tinker, Freya Probst, Sebastien Ourselin, Michela Antonelli, Erika Molteni, Nikitia Mexia, Yu Shi, Faith Matcham

**Affiliations:** a Department of Global Health and Social Medicine, King’s College London, London, UK; b Department of Engineering, King’s College London, London, UK; c School of Psychology, University of Sussex, Falmer, UK; d School of Biomedical Engineering & Imaging Sciences, King’s College London, London, UK; e School of Design, University of Leeds, Leeds, UK

**Keywords:** Loneliness, older adults, qualitative, technology, lived experience, public engagement

## Abstract

**Purpose:**

Loneliness is a negative emotional state which is common in later life. The accumulative effects of loneliness have a significant impact on the physical and mental health of older adults. We aim to qualitatively explore the experiences of loneliness in later life and identify relevant behaviours and indicators which will inform novel methods of loneliness detection and intervention.

**Methods:**

We conducted 60 semi-structured interviews with people aged 65 and over between September 2022 and August 2023. Data were analysed using a reflective thematic approach with early theme development on NVIVO software.

**Results:**

Three themes were identified from the experiences of loneliness in older adults. 1) Unique responses to loneliness, including crying, increased eating or drinking and sleep difficulties, 2) Age-related losses, such as networks, roles, and abilities to engage in activities reducing over time and 3) Individual differences in overcoming loneliness, where strategies such as keeping busy and adopting a positive mindset were impacted by motivation and mood of older adults.

**Conclusion:**

Distinct signs and relevant factors to loneliness in later life have been identified which can be detected by future sensing technologies. Findings of this in-depth qualitative study highlight that loneliness is a subjective experience requiring a holistic and person-centred approach to detection and intervention.

## Introduction

Promoting health and wellbeing in the elderly is a global priority to address the challenges of an ageing population (World Health Organisation, 2023). Loneliness is a subjective negative emotional experience which is common in later life, affecting around one in four older adults (Chawla et al., 2021). Globally, loneliness is at a problematic level (Surkalim et al., 2021). In the UK, research estimates around three million people over the age of 75 report feeling lonely often or always (GOV.UK, 2021), a figure predicted to have grown as a consequence of social isolation during COVID-19 (Chatzi & Nazroo, 2021). According to evolutionary theories, loneliness is an innate signal to motivate behaviour change and encourage connection with others (Cacioppo et al., 2014). However, the accumulative negative impacts of loneliness can cause an adverse effect on an individual's health status (Courtin & Knapp, 2017; Hawkley & Cacioppo, 2010; Martín-María et al., 2020). In older adults, studies have consistently shown the relationship between loneliness and increased mortality (Holt-Lunstad et al., 2015; Shiovitz-Ezra & Ayalon, 2009) due to impacts on the cardiovascular system, health behaviour and mental health (Coyle & Dugan, 2012; Hodgson et al., 2020; Leigh-Hunt et al., 2017; Valtorta et al., 2016). Such evidence highlights how loneliness is an important public health issue to address (The Lancet, 2023). To facilitate appropriate support for ageing communities, it is critical to understand the interaction between an individual’s social needs and health challenges (Wotherspoon, 2023).

The severity, pervasiveness and chronicity of loneliness varies across individuals and time (Lim et al., 2020). Categorization of loneliness severity (i.e., not lonely, moderate, severe) is achieved through use of validated measures such as the De Jong Gierveld Scale (De Jong Gierveld & Tilburg, 2010) or the University of California, Los Angeles (UCLA) scale (Russell, 1996). Those who report feeling lonely most or all of the time are defined as experiencing chronic loneliness, whereas short-term loneliness is defined as transient (Young, 1982). Social loneliness is conceptualized as arising from a discrepancy in desired and actual social contacts and connections (Perlman & Peplau, 1981). Emotional loneliness refers to the absence of meaningful relationships (Mansfield et al., 2021). To address these core mechanisms of loneliness, recent models (Akhter-Khan et al., 2022) have sought to identify the contextual factors related to ageing, for example health decline, loss of networks and shifting roles to enhance understanding of what older adults expect from social relationships.

Technological advances offer promising solutions to support older adults experiencing loneliness. In the field of loneliness intervention, detection and prediction technologies are emerging. Such approaches operate on the assumption that changes in certain behaviours can be recorded and used to infer the onset of loneliness or even its severity. To date, detection and prediction approaches have focused on behavioural indicators of loneliness. Homes equipped with smart technologies can enable the passive monitoring of activities indicative of such behaviours (Latikka et al., 2021). The use of mobile sensing through smartphones and wearable devices, for example using fitness trackers, has also been utilized to monitor a variety of mental and physical activities relatable to behaviours dictated by loneliness states. However, this research has largely been conducted in younger populations (Qirtas et al., 2022). Preliminary research has demonstrated the potential to identify levels of loneliness in older adults through the collection and monitoring of multiple objective behavioural measures such as phone use and out-of-home activity (Austin et al., 2016; Petersen et al., 2015). In addition to being a cognitive and emotional phenomenon, feelings of loneliness produce a somatic response in an individual; thus, loneliness has a physical dimension (McKenna-Plumley et al., 2023). In stress research, sensor-based technologies have been used to continuously monitor physiological data such as heart rate, skin temperature, blood pressure and respiration rate, to detect early signs of stress (Gedam & Paul, 2021). A gap remains for future research to explore the use of such technologies to identify the state and severity of loneliness in older adults.

The “Design for Healthy Ageing: a smart system to detect loneliness in older people” or DELONELINESS project aims to address the gap in the literature on the use of sensor-based technologies aiming to develop a smart monitoring and communication system with multi-functional electronics built into textiles used as wearables and home furniture to detect and measure loneliness levels in people aged 65 and over (Rees, Matcham, et al., 2023). However, prior to the development of such technologies, more information is needed about the signs and experiences of loneliness in later life, which need to be prioritized for measurement. There is a scarcity of high-quality data in this population. Qualitative methodologies enable a unique understanding into the subjective and idiosyncratic lived experiences of loneliness. To achieve our objective of exploring “older people’s experience of loneliness across a number of psychological and social parameters and behaviours” (Rees, Liu, et al., 2023), we analysed qualitative interview data to answer the following research question: What are the relevant signs and behaviours associated with loneliness in later life?

## Methods

Methods of this study have been detailed in the study protocol (Rees, Liu, et al., 2023). Ethical approval was obtained by the Research Ethics Committee at King’s College London (reference number: LRS/DP-21/22-33376) and the University of Sussex (reference number: ER/JH878/1).

### Participants

Semi-structured interviews with people from the United Kingdom aged 65 and over were conducted between September 2022 and August 2023. Older adults who self-identified as having experienced loneliness since reaching the age of 65 were purposively recruited via involvement in previous research projects, organization newsletters, and research participation websites. Interested individuals were invited to contact the research team to obtain further details. After receiving the study information sheets, two older adults were not happy to participate as the interview recordings were being sent to a transcription service, and one older adult was ineligible following a capacity assessment. Once interviews were organized, one participant dropped out due to a bereavement and one dropped out due to recent experience with scam. All participants provided written informed consent prior to data collection.

### Procedure

The lead author (a female post-doctoral researcher and chartered psychologist with expertise in sensitive interviewing) conducted interviews in a variety of formats based on the preferences of participants. These included face-to-face, at home or in university offices, by telephone, or video call using Microsoft Teams software. To account for the interdisciplinary nature of the DELONELINESS project, the researcher positioned themselves as a psychologist at the beginning of interviews and reiterated to participants the importance of hearing the lived experience of older adults for the project future objectives. For 15 out of 60 interviews a psychology student was present to shadow the researcher. The purpose of this was to provide a learning opportunity for undergraduate students in qualitative interviews and was conducted with participants’ consent. Students only observed online interviews, and after being introduced to the participant, kept their microphones on mute and camera off for the duration of the interview to minimize any observer effects.

Prior to the interview, participants answered a series of questions verbally to the researcher including sociodemographic information, social environment, and medical history. To determine current loneliness, mood and health service use at the point of data collection: for loneliness we included the De Jong Gierveld 11-item Scale (De Jong Gierveld & Tilburg, 2010) and UCLA Loneliness 4-item scale (Hughes et al., 1999); for mental health we included the Patient Health 4-item scale for anxiety and depression (Kroenke et al., 2009, 2016); and for health service use included the Modified client Service Receipt Inventory (Chung et al., 2021). As a relationship was not developed between the researcher and participant prior to study commencement, the questionnaire completion offered an opportunity to develop rapport.

The first section of the interview focused on participants experience of loneliness in later life. See Appendix 1 for full topic guide. Based on guidance for conducting ethical interviews on loneliness with older adults (Naughton-Doe et al., 2022), the interview began with an open-ended question for participants to provide their own definition of loneliness. The interviewer used responses to tailor language based on participants personal definition. Follow-up questions focused on participants experiences of loneliness with prompts used from responses to the validated loneliness measures. We asked about the impact of loneliness on mental health, physical health, social life, and relationships, in addition to precursors to loneliness and strategies to reduce feelings of loneliness. The section of the interview related to loneliness lasted between 30 and 60 min. To provide further details for smart system development, the remaining interview questions focused on daily routine, home environment, existing use of technologies, preferences for sensors, thoughts on usefulness of data, and involvement of family members and/or healthcare professionals.

Notes on participants’ responses to questions were made by the researcher to facilitate the development of prompts throughout the interview. At the end of the one-off interview, participants were provided with a £30 voucher as a thank you. Interviews were audio recorded and transcribed verbatim by a professional company.

### Public involvement

To include the voice of older adults with lived experience, we incorporated public involvement strategies throughout the study. Preliminary findings were discussed in two meetings with the DELONELINESS study public advisor (LC) with insights from this meeting used to refine specifics of each theme. Although transcripts were not returned to participants for comment or correction, results were shared as a lay summary for feedback from those who consented to be contacted about the findings.

Our recruitment strategy was reviewed by a multicultural public involvement group specially trained to advise on research. Variations in cultural conceptualizations of loneliness were highlighted in this meeting. We therefore conducted three workshops with people from minority ethnic groups to increase the transferability of findings to lesser-represented populations (Rees, 2023). Older adults in these workshops recognized and were familiar with the experiences of loneliness described by the DELONELINESS sample. Similar fears and anxieties about living alone were described in addition to the loss of parental purpose and the impact of families living far apart. Unique themes included the benefits of religion in alleviating loneliness by providing a positive mindset, a sense of purpose and routine. Workshop attendees also described how language proficiency acted as a barrier to connection in the local community increasing social isolation.

### Analysis

Data were analysed using a reflective thematic approach with structured early theme development (Braun & Clarke, 2019). We began with a codebook of signs (physical, behavioural, emotional and cognitive) as outlined in previous research on stress (Gedam & Paul, 2021) and loneliness (McKenna-Plumley et al., 2023). We used this codebook based on literature evidence as an initial structure (see Table II) and inductive codes were organized underneath each sub-theme using NVIVO-14 software. The lead author adopted an inductive approach throughout the analysis to identify patterns of meaning across the data. For example, adding additional themes to the codebook (age, health, relationship, social factors) during the theme generation phase.

Following familiarization with transcripts, the lead author coded interview data where participants described an experience, behaviour or associated psychosocial factor related to loneliness. Initial codes were then reviewed to ensure they were categorized under the appropriate theme. The ongoing process of defining and naming themes was facilitated through discussions with a public contributor (LC) with lived experience of loneliness. In these discussions, a summary of the findings was presented with feedback facilitating the refinement of the codebook into the finalized themes.

To inform our contextual understanding of the experiences of loneliness in later life, we cross-referenced all risk factors for loneliness identified in our evidence-based conceptual model (for example, depression, mobility, loss) to codes identified in interview analysis (Rees, Liu, et al., 2023). To increase reliability, a second researcher (JC) coded a random selection of interviews (*n* = 10) to review candidate themes. Similarities and differences in coding and theme interpretation were discussed with the lead researcher.

## Results

### Participant characteristics

Data were collected from 60 participants with a mean age of 73, ranging from 65 to 91 years old. The majority of participants were aged between 65 and 75 years (*n* = 42), female (*n* = 42), white British (*n* = 54), homeowners (*n* = 43), and degree educated (*n* = 34). See Table I for full details.

**Table I.** Characteristics of participants.

Age	65-75	42 (70%)
	75+	18 (30%)
Gender	Female	42 (70%)
	Male	18 (30%)
Ethnicity	White/White British	54 (90%)
	Black/Black British	3 (5%)
	Asian/Asian British	3 (5%)
Education	Degree	34 (57%)
	A Level	10 (17%)
	GCSE	9 (15%)
	No qualification	7 (11%)
Accommodation type	Own home	43 (72%)
	Rented	10 (17%)
	Sheltered accommodation	7 (11%)

Marital status varied from Widowed (*n* = 20), Divorced (*n* = 16), Single (*n* = 11), Married (*n* = 8) and Separated (*n* = 5). Thirty per cent of participants did not have children (*n* = 18) and 13% did not have anyone to contact if they were in trouble of needed help (*n* = 8). The majority of participants reported having a long-standing illness or disability (*n* = 50) such as depression, chronic fatigue, arthritis or hearing loss. Based on the analysis of the De Jong Gierveld Scale, the severity of loneliness in our sample varied from Very Severe (*n* = 11), to Severe (*n* = 10), Moderate (*n* = 36) and Not Lonely (*n* = 3). The frequency of loneliness also varied from Often/Always (*n* = 23), Occasionally (*n* = 16), Some of the time (*n* = 20) and Never (*n* = 1). Twenty-three per cent participants (*n* = 14) screened positive for Major Depressive Disorder by scoring higher than a 3 on the Patient Health Questionnaire 2-item scale, while 42% participants (*n* = 25) screened positive for generalized anxiety by scoring higher than a 3 on the Generalized Anxiety Disorder 2-item scale.

### Themes

Three themes were identified exploring the lived experience of loneliness in older adults. 1) Unique responses to loneliness, 2) Age-related losses, and 3) Individual differences in overcoming loneliness. See Appendix 2 for full list of supporting quotes.

### Theme 1: Unique responses to loneliness

One sign of loneliness described by participants was crying, which ranged from feeling tearful to sobbing. Crying was associated with intense emotions of sadness, feeling miserable or down, a sense of despair or gloom. Such emotions could be “set off” by external factors such as watching television or occurred for no particular reason, however this sign always occurred when they were on their own.

I felt like crying, I felt like crying through the loneliness, feeling lonely I felt like crying. I just felt like I was going to go up the wall living on my own. (P55, Female, 73, Single, Moderate Loneliness)

A key factor related to feelings of loneliness was lack of companionship. Several participants described using the noise from the television or radio to break up the silence of not having anyone to talk to for older adults living alone. Emptiness was a sign felt by older adults as a hollow sensation, an ache in the chest or a feeling in the pit of the stomach. Participants described feeling empty all the time (i.e., chronic loneliness) or at specific times such as returning home from an activity or seeing others (i.e., transient loneliness). To manage such feelings, older adults described increased eating and/or drinking, which occurred primarily in evenings and/or on weekends. Male participants referred to drinking as a “great companion” which could “take the edge off” while female participants spoke about binge or comfort eating sugary foods.

I think one thing we didn’t touch on which really I should touch on is that I do drink a lot of alcohol. That might be worth putting in the notes as to whether that makes me less lonely or more lonely. (P22, Male, 83, Widowed, Moderate)

It’s an inner pain. Sometimes it feels like an enormous weight. Sometimes it feels like an enormous hole. And I feel desperate to fill it and to be comforted and I do tend to eat more than I should for that reason. (P18, Female, 70, Married, Very Severe Loneliness)

Sleep difficulties, having trouble going to sleep or having disturbed sleep, were another frequently described indicator related to loneliness. In addition to being associated with age-related health factors such as an overactive bladder, sleep difficulties were also associated with overthinking and rumination. Older adults described worries going “around and around” in their head and thinking about worse case scenarios (i.e., catastrophising). Participants discussed thinking about conversations they had with others “for days” in their mind and would ruminate on “stupid” things they said or did in social situations which worsened their feelings of loneliness. Older adults would also compare themselves when they saw how others were spending time with loved ones or taking part in enjoyable activities. For example, asking “what if I had a partner?” or “why can’t I be sociable like that?” This left one participant feeling like an “observer rather than a participant in life” increasing their sense of isolation from others.

And that’s I think when I question and think about things or if I feel everybody else is having fun and I’m not type of thing that’s a difficult one. I do feel lonely then. (P13, Female, 69, Separating, Moderate)

It’s very much rumination and it just goes on and on … it’s always catastrophes and disasters. You know, it’s all awful and everybody in the world hates me and all that sort of thing … I think the thoughts make the feeling of loneliness worse … it’s all so tied up with my self-image that it is, it’s that that is the worst part of the whole thing really I think. (P18, Female, 70, Married, Very Severe)

### Theme 2: Age-related losses

Loss was found to be a significant factor associated with loneliness in later life. Participants spoke about their network reducing or changing over time due to death, illness or relocation. Such recognition reminded older adults of their own mortality (“last chance saloon”) which caused negative feelings (i.e., regret, mourning) as they reflected on their past life and social connections. Several participants highlighted the difficulties in making new friends as they got older as people “get set in their ways” or it was difficult to make the “first move” to initiate conversation.

So it’s hard to make, you know, to sort of, to get friendship when people can do things that you’re not able to do… Because I can’t say to them, would you like to do this, that and the other, because I can’t do things anyway…So that is why I am so lonely (P46, Female, 87, Widowed, Moderate)

Participants described how loss of physical and emotional intimacy impacted feelings of loneliness. Older adults valued contact with grandchildren, specifically hugs, a connection which provided a “thunderbolt” or “hormonal buzz” feeling. However, relationships with family were acknowledged to change over time (“he didn’t want to come here anymore”). Similarly for widowed participant, connection which was lost from the death of their spouse were not replaceable and contributed to feelings of loneliness. Widowhood also created a loss of “unshared experiences” which came from a lack of intimacy in sharing situations with another.

As children grew up or since retirement, older adults described experiencing a loss of purpose as changing roles meant they felt not needed anymore. Despite retiring in their mid to late 50s and early 60s, participants described still adjusting to not seeing work colleagues or being busy with full time work (“go from that to nothingness”). As a result of such losses of identity, older adults found themselves questioning the point in living as they felt life was “pointless.”

Not being able to give is just awful. Not to have a purpose. Not to have a relevance. Not to be, not to be of any interest to anybody, I think it’s just, it’s just, it’s just a horrible thing. (P21, Male, 73, Married, Moderate)

Loneliness was found to worsen in later life as participants were unable to do the same activities due loss of functional ability through physical deterioration and fear of injury. Mobility issues, pain and fatigue from health conditions impacted older adults ability to socialize in ways they would have previously done. Many participants lived with conditions which were described as “debilitating” as they impacted day-to-day activities (e.g., going up the stairs, shopping), enjoyable hobbies (e.g., gardening, hiking), and connection with others (e.g., feeling understood, ability to converse). The latter point was especially relevant to older adults with hearing impairments.

So I notice more about people’s, what they do with their hands and their faces and things like that because I can’t quite hear what they say. And there’s a limit to the number of times you can say, Could you say that again? So, it is very, very isolating. (P38, Male, 80, Married, Moderate)

### Theme 3: Individual differences in overcoming loneliness

Older adults highlighted how they would “keep busy” to distract themselves from feelings of loneliness or engage in meaningful activities which brought “pleasure and joy.” Participants joined activities and hobbies (i.e., lectures, exercise classes, volunteering) as a way of meeting people. Older adults with severe loneliness reported finding it hard to relate to others and form in-depth connections with people as they did not feel “understood” and contact “rarely lasted” beyond organized activities. Male participants also expressed being less able to organize activities among peers or speak about emotions and feelings, which may have been a barrier to establishing and maintaining deep relationships to overcome emotional loneliness.

Have a regular meeting and they get together. Fantastic, they support each other brilliantly. Women are much better at doing that and keeping those connections than men. So that I feel, I can be lonely here in the house. (P21, Male, 73, Married, Moderate)

The ability to engage in activities to reduce loneliness depended on motivation of older adults. Feelings of low mood caused participants to withdraw as they did not have the “oomph” to reach out and did not want to “burden” or “inflict” themselves on others. Many older adults discussed the bi-directional relationship between depression and loneliness. Participants highlighted how both were separate emotions that were also connected. This led to a “self-perpetuating” cycle where older adults both wanted to do something about loneliness but lacked the energy to do so. Older adults with clinical depression were found to have greater negative thoughts about themselves (e.g., feeling worthless, unattractive) and others (e.g., no one is interested, no one loves me). Such perceptions impacted the mood of older adults, specifically self-worth, and made them hesitant to engage in the future. However, people with moderate loneliness described being able to reach out to friends or family members where they had not heard from them in a while.

The psychological thing is feeling worthiness. I think to me that is important, to reinforce the self-worth … Yes, it’s not worthy, no one can be bothered with you blah blah blah. (P49, Female, 72, Divorced, Severe)

There is nothing like overcoming your loneliness through engaging with others in a way that you can give and if you can give, you recover your self-esteem as well. (P23, Male, Separating, Not Lonely)

Some participants expressed the importance of “doing something” about loneliness as they recognized it was “down to them” to change behaviour. Interestingly, male participants mostly expressed “soldiering on” due to a sense of “having to be okay” to avoid being a burden on family or friends. Older adults described loneliness as a “part of life” they learnt to cope with by getting on “with what you have to do” to maintain health and wellbeing. Some participants felt it better to not “wallow” in feelings and focus on positive aspects (i.e., what you have rather than don’t have) suggesting how personality characteristics may influence older adults ability to cope with loneliness.

Well I just think it depends on your personality, how you cope with it, you know. Some people sort of moan and whatever and they won’t do anything about it, you know. And I often say, well why don’t you go and do this or join this or whatever, you know. And you know they moan and groan and I just think, well no, I mean, that’s not me. (P45, Female, 81, Single, Moderate)

## Discussion

This study used a qualitative approach to explore the lived experience of loneliness in later life to identify the associated indicators and behaviours. Our future research will incorporate these findings into the design of a smart system to detect and measure loneliness in this population and explore older adult preferences for loneliness technology development (Rees, Matcham, et al., 2023). For the present study, three themes were identified. The first theme reflects the unique and personal experiences of loneliness by exploring the similarities and differences in the subjective reactions of older adults in response to loneliness. Many of these signs and associated behaviours were expressed when participants were on their own, suggesting an alignment with social loneliness (De Jong Gierveld & Tilburg, 2010). Living alone is common in later life and well referenced as a risk factor for loneliness (Klinenberg, 2016). However, other participants in our study lived with family members and/or spouses, confirming the need to broaden conceptualizations of loneliness in later life. Authors have argued that loneliness is a structural condition based on social narratives which focus on families and couples, creating a sense of personal failure if this is not achieved or maintained (Wilkinson, 2022). These findings highlight the importance of considering sociodemographic factors (i.e., martial status, accommodation type) of older adults when designing future technologies to support healthy ageing. Such factors have previously been identified as risks or correlates in conceptual models of loneliness (Lim et al., 2020).

The physical dimension of loneliness, for example anxiety responses, numbness, and tightness in the chest, has previously been described in a general synthesis of loneliness features across the life course (McKenna-Plumley et al., 2023). Our findings on the physical and mental experiences of loneliness in people aged 65 and over emphasize the embodied experience of loneliness (Bound Alberti, 2018). We expand this knowledge further by combining the indicators of loneliness with associated behaviours (outlined in Table II). Many of the associated behaviours identified in our study have potential implications for the health of older adults experiencing loneliness. Increased alcohol consumption and food intake are examples of health-limiting coping strategies adopted by older adults during COVID-19 (Finlay et al., 2021). International qualitative studies from Finland and New Zealand have found reasons for alcohol consumption in older adults to be related to feelings of loneliness (Immonen et al., 2011; Khan et al., 2006). In future remote detection technologies, it would be important to ensure the individual indicators identified (e.g., crying, increased eating or drinking, worry) were specific to loneliness and could be differentiated between related conditions such as depression or stress. Loneliness has been suggested to be an “emotion cluster” as it includes multiple, interacting, complex emotions (Bound Alberti, 2018). The association between loneliness and depression is well established in older adults, thus strategies to reduce loneliness could also reduce depression (Lee et al., 2021).

**Table II.** List of indicators and behaviours associated with loneliness in later life.

Dimension of loneliness	Indicators and associated behaviours
Physical	Crying (tearfulness, sobbing) Ache in chest Feeling in pit of stomach Sleep difficulties Lack of physical contact
Behavioural	Increased eating or drinking Watching television or listening to radio Unable to do same activities (mobility, pain, fatigue) Keeping busy Lack of motivation
Emotional	Emptiness Sadness, feeling miserable Sense of despair or gloom Worry Low mood
Cognitive	Overthinking and rumination Negative thoughts about self Comparison with others Reflecting on past life Questioning point in living Focus on positives

Further evidence for the psychosocial factors of loneliness was described in the second theme which reflected the broad spectrum of age-related losses associated with loneliness. Later life is well recognized as a time of transition (Nilsson et al., 2000). Losses described by participants in our study included networks reducing over time, changing roles in families and work, and physical deterioration. Two social relationship expectations for older adults relevant to our findings are intimacy and generativity (Akhter-Khan et al., 2022). The former relates to the emotional dimension of loneliness (De Jong Gierveld & Tilburg, 2010) and is the expectation of older adults to feel close, listened to and understood by loved ones. The latter refers to the expectation of older adults to be able to meaningfully contribute. The World Health Organization defines healthy ageing as “the process of developing and maintaining the functional ability that enables wellbeing in older age” which includes being mobile, being able to build and maintain relationships, and being able to contribute to society (World Health Organisation, 2022). Qualitative work from Sweden has identified the need to feel useful as central to a sense of psychological well-being in older adults in addition to “feeling well” despite health concerns (Gillsjö et al., 2021). Our findings highlight how participants’ ability to engage in activities which prompted social connection were limited by their functional abilities, for example pain and fatigue, both commonly reported signs in people with loneliness (Powell et al., 2021). A recent qualitative study highlighted how health challenges can impact loneliness with indicators restricting ability to socialize and stigma leading to social withdrawal (Wotherspoon, 2023). Older adults in our study described how health conditions impacted their ability to connect with others, further evidencing the complex interactions between poor health and loneliness (Dahlberg et al., 2022; Shi et al., 2023). These findings highlight the importance of understanding the lived experience of loneliness to consider relevant health and social factors associated when designing future sensing technologies to support wellbeing in later life.

Our final theme described the individual differences for engaging in behaviours or activities to alleviate loneliness. Our findings highlight gender differences in coping with feelings of loneliness. In a qualitative study on men’s perspective of loneliness, the notion of “busy” was frequently associated with being not lonely and how engagement in mentally stimulating activities acted as a “bridge” to forming social connections with others (Ratcliffe et al., 2023). Participants in our study either felt they had to do something about loneliness themselves or struggled to engage in activities and form deep connections with others. Being willing or feeling able to speak about emotions has previously been described as a barrier for men in forming intimate and supportive connections (Ratcliffe et al., 2023). Almost a third of interview participants screened for Major Depressive Disorder, a factor associated with low self-worth and severe loneliness (Cohen-Mansfield et al., 2016; Courtin & Knapp, 2017). A recent cognitive behavioural theory proposed that negative interpersonal appraisals (i.e., people will not like me) lead to counterproductive behaviours (i.e., withdrawal from social interactions) and negative emotional responses (i.e., sadness, anxiety) perpetuating the cycle of chronic loneliness (Käll et al., 2020). Our findings highlight how negative appraisals were common in older adults with chronic and severe loneliness, which impacted ability to engage in activities to reduce loneliness.

Psychological approaches to overcoming loneliness aim to change how an individual thinks and feels about their social connections (Campaign to end loneliness, 2020) also referred to as maladaptive social cognitions (Masi et al., 2011). Despite evidence for the effectiveness of psychological approaches in improving loneliness for older adults, recent reports suggest that they have yet to be implemented in practice (DCMS, 2023). Researchers have concluded that a one-size-fits-all approach to overcoming loneliness is not appropriate or effective (Victor et al., 2018) and a personalized approach to loneliness intervention is required (Akhter-Khan & Au, 2020). Our findings highlight the individual signs, context and coping of older adults in response to loneliness, which can be used to tailor technology-based precision health approaches. Future research can use findings related to the barriers to motivation faced by this population to inform holistic approaches to intervention. For example, our findings may have implications for the initiation and engagement with interventions to overcome loneliness.

### Strengths and limitations

This study adds to the existing literature on the lived experience of loneliness in later life with a novel focus on identifying relevant indicators and behaviours to inform the design of future technology developments. Strengths of this study include a large sample from a wide geographical area to explore loneliness in a varied population of older adults. We purposively recruited a range of older adults to participate in individual in-depth qualitative interviews in terms of age, gender, location across the UK, accommodation type and level of digital ability. We endeavoured to collect data from a range of living environments; however, we did not collect reportable data on the location of participants. Our future work will explicitly measure and report on the urban or rural location of participants to account for the impact of such settings on experience of loneliness.

Despite purposefully targeting an equal distribution of male and female participants, we still recruited a majority female sample. The under-representation of men in mental health research is a well-documented phenomenon (Woodall et al., 2010) potentially exacerbated in our study by the stigma associated with loneliness in men (Ratcliffe et al., 2021). By recruiting individuals who self-identify as lonely, we may have inadvertently created a barrier for participation for men who may be less likely to identify as lonely (Ratcliffe et al., 2024). This contributes to our findings representing a female-dominant perspective; different themes may have emerged if greater gender balance had been achieved.

Using validated questionnaires, we identified the proportion of our sample which screened for clinical depression and anxiety. To enhance methodological rigour, we followed the consolidated criteria for reporting qualitative research (Tong et al., 2007) and utilized feedback from public involvement through the study process. A limitation of our study was the lack of ethnic diversity, with the majority of our sample being White British. To overcome this limitation, we validated our findings in workshops with older adults from minority ethnic groups (Rees, 2023). Our recruitment sources may have also been biased towards older adults with existing access to support from engagement in previous research studies or by accessing housing services. As such, our findings have limited generalizability to older adults living in greater isolation than the population recruited in our sample.

## Conclusion

The negative effects of loneliness can accumulate over time, creating implications for the health and wellbeing of older adults. As such, early detection of loneliness is paramount to promote healthy ageing and enable timely access to support for this population. Findings of this in-depth qualitative study highlight how loneliness is a subjective experience with distinct signs that may be measurable by a smart detection system. Themes explored the unique and personal reactions to loneliness in later life, age-related losses, and barriers to engaging in activities to alleviate loneliness. Future research should explore person-centred approaches to tackling loneliness in later life following identification using sensor-based technologies.

## Appendices

### SEMI-STRUCTURED INTERVIEW

*Ethical Clearance Reference Number*: LRS/DP-21/22 -33,376

### Exploring the Psychological Experience of Loneliness

The primary purpose of this interview is to gain insight into the loneliness in older people, and to inform sensor development work being conducted within the DELONELINESS project. The purpose of this interview is threefold:

1. identify the psychological and social parameters of loneliness, prioritized for consideration within a smart system;
2. describe the context and circumstances in which a smart system might be most useful; and
3. identify the most meaningful way of providing information back to individuals, their carers, or healthcare/social service providers.

Anonymized quotes may be used in internal reports, external publicity (such as soundbites on the DELONELINESS website), and for research purposes. The interview will be a maximum of 2 h, and will be recorded for future reference. This guide is designed to provide a structure for interviewers to follow, but does not rule out opportunities to adapt or change the questions, or their order, depending on what the interviewee says.

**Table:**

**Interview Phase/Purpose**	**Questions/Prompts**
“Thank you for participation in this interview. I’d like to start by asking you some questions about your experiences of loneliness, either now, or in the past. For all of these questions, please try and specifically think of a time you’ve felt lonely since your 65^th^ birthday”	
**Definition of loneliness**	Can you tell me how you describe loneliness? What does the word mean to you? Do you identify with another word? (social connection, social isolation, aloneness, solitude).
	*Prompt* Social loneliness (discrepancy between actual and desired quantity and quality of social interactions, includes cultural differences). Emotional loneliness (absence of meaningful relationships, negative feels can occur ever in close contact with people). Existential loneliness (sense of separateness from others and wider world, particularly being illness and bereavement). Relationship-specific loneliness (romantic partner, siblings, children, friends, community).
**Confirming experience of loneliness** Identify when the individual felt lonely	You mentioned in the questionnaire that [insert answer from loneliness measures]. Thinking about these aspects can you tell me: 3. Please describe the last time you felt lonely? 4. How long did you feel lonely for?*Prompts: Did you feel lonely for weeks or months or years? Or for hours and days? Different before/after COVID?* 5. How long ago were you feeling this way, or are you still feeling lonely? 6. Did you feel lonely all the time or was it broken up by periods of not feeling lonely?
**Personal experiences of loneliness**	7. When you think about the most significant period of loneliness you’ve experienced since your 65^th^ birthday, can you describe how it felt?
	*Prompts: Describe anything that comes to mind – your mental health, social life, relationships, physical health…*
**Correlates of loneliness** Establish the holistic experience of loneliness.	“Thank you for sharing that with me. You mentioned that [insert answer to Q7] was associated with the loneliness you experienced. Can you tell me more about that? *Prompt: Did they occur at the same time? Can you describe the experience in more detail? Did X finish when the period of loneliness had ended?* Repeat for all the things listed for Q7…
**Precursors to loneliness** Determine what things might be able to predict loneliness.	Thinking about the most significant period of loneliness you’ve experienced since turning 65… 8. Can you remember anything happened in the weeks, months or years leading up to it which might have contributed to it?
	*Prompts: Had you had any changes to your usual routine, life events, or diagnoses? COVID-19?* “You’ve mentioned that [insert answer to Q8] happened shortly before your most significant period of loneliness.”
	9. How long, roughly, before you started feeling lonely, did you start to experience this? 10. Is there anything that could have stopped [insert answer to Q8] from influencing your loneliness? 11. Is there anything else you haven’t mentioned so far that could have been associated with the onset of your most significant experience of loneliness?
	*Prompts: Feel free to mention anything, no matter how big or small it might have seemed at the time.*
**Implications of loneliness and support received** Understand what the end of a loneliness event might look like and the care pathways used.	12. Thinking about your most significant period of loneliness, can you tell me more about the aspects of your life it affected?
	*Prompts: Describe anything that comes to mind – your mental health, social life, relationships, physical health…* 13. How did the loneliness end? Did it finish naturally, or was there an event or intervention which helped it go away? 14. If something specific helped it go away, can you explain what happened?
	*Prompts: Did you actively seek help? Who did you speak to? What did they do? If you didn’t actively seek help, what triggered the change in your loneliness*.
	15. If your loneliness is ongoing, what do you think would help it go away?
**Final thoughts** Opportunity to discuss anything else	“Is there anything else about your experience of loneliness which you would like to share before we move onto the next part of the interview?”
**OPPORTUNITY FOR A BREAK IF REQUIRED**	
**The role of technology in measuring loneliness** “Thank you for sharing such personal information about your experiences of loneliness. The next part of the interview is going to move on to talk about who technology might be used to help us measure loneliness more effectively.”	
**Environment and daily routine** Establishing patterns of activity when lonely and when not lonely	“I would like to move on by asking some questions about your daily routine and your living environment.”
	1. Describe your living environment to us please? 2. Which room/furniture in your living environment do you use the most? How long?
	*Prompts: Are you living in a flat or a house? How many rooms are there? Do you have regular house guests?*
	3. Can you describe a typical daily routine for you?
	*Prompts: What time do you tend to wake up? Do you have the same breakfast every day? Do you leave the house every day?*
	4. Does this routine change much when you’re experiencing loneliness? 5. [If yes] What tends to change in your daily routine when you’re lonely?
**Existing use of technologies**	6. Do you use technology to measure any aspect of your health right now?
	*Prompts: This could be an app on your phone, a website you access regularly, or some kind of technology that you wear or carry with you to measure something*.
	7. [If yes], please tell me more about it.
	*Prompts: How often do you use it? What works well/favourite? What works less well/least favourite?*
**Data collection requirements** Identify priorities for the smart system	“Part of the DELONELINESS study is developing new ways of measuring loneliness, through sensors which might be worn on the body, or integrated into a fabric. These sensors will measure things, such as heart rate, or small movements you make, which might be a useful indicator of loneliness.”
	8. Thinking now about a device which you might wear on your body, where on your body would you be willing to wear a device?
	*Prompts: Would you be willing to wear something around your wrist like a watch? Would you prefer something on your ankle, or attached to your chest?*
	9. Wrist: what would make you less willing to wear this device every day? What would make you more likely to wear this device every day?
	10. Would you be willing to wear the device all day, or would you want to be able to take it off sometimes? Why would you want to take it off?
	Another option might be to have a sensor which is in fabric in your clothes, or in furniture.
	11. If we were to integrate the sensor into fabric in a piece of furniture, what would be the most convenient piece of furniture to use? 12. What would make you less willing to use this piece of furniture every day? What would make you more likely to use this piece of furniture every day? 13. If we were to integrate the sensor into fabric in an item of clothing, what type of clothing would be most useful? 14. What would make you less willing to wear this item of clothing every day? What would make you more likely to wear this item of clothing every day? 15. Would you rather have clothing which had a sensor in, or would you rather have a piece of furniture with the sensor in? 16. What types of materials or fabric bring you the most comfort at home?
**Loneliness impact on engagement** Establish whether answer to any of these questions might be different when lonely.	Thinking about the conversation we’ve just had about your preferences for wearable technologies and textile sensors. 17. Do you think any of your opinions or requirements would change if you were feeling lonely? 18. If you’re currently feeling lonely, do you think any of your opinions or requirements would change if you weren’t feeling lonely right now?
**Final thoughts** Opportunity to discuss anything else	19. Is there anything else about your preferences or requirements for a device which you haven’t had an opportunity to mention so far?
**OPPORTUNITY FOR A BREAK IF REQUIRED**	
**1. Data feedback and integration into services** “Thank you for giving us an insight into your living environment and your preferences for how we could be measuring loneliness using sensors. We’re now going to move onto the final part of the interview, which focuses on what we should do with the data we collect. We might be able to collect data about a wide range of things, such as your sleep, movements and stress levels.”	
**Data recipients** Who should receve it?	20. How useful would this data be for you, your family or caregivers and your GP to receive? 21. Is there any other person or service this data could be sent to help improve your quality of life?
**Implications of data** What the data would mean	22. If this data were sent to you…what benefits are there to having this kind of information available?
	*Prompts: Might this data change your daily routines, or prompt you to do something different?*
	23. What limitations might there be to having this kind of information available?[repeat questions for family or caregiver and GP if relevant]
**Data requirements and actions** How data might be received	24. What would you do if we were to alert you that you were at risk of becoming lonely? 25. If this data were sent to you…how would you want to receive it? How often? 26. What would you hope a carer or family member/GP would do if we were able to alert them that you were at risk of becoming lonely?
**Final thoughts** Opportunity to discuss anything else	27. Is there anything else about your what we could do with the information we collect you haven’t had an opportunity to mention so far?

## Appendix 2. List of supporting quotes per theme

**Table:**

Theme	Supporting quotes
Unique responses to loneliness.	*Crying* “So, sad that one feels lonely … Physically, yes, everyone gets lonely, one could have a bloody good cry.” (P40, Female, 75, Single, Moderate Loneliness). *Watching television* “I’ve been watching the television too much, and that makes a noise, or the radio will make a noise, so, you know, hearing voices or music can help to feel that you’re with someone because- well, it’s- sometimes, I can go all day, several days together, and the only voice I hear is mine.” (P48, Female, 86, Single, Moderate) *Increased eating* “Never in the day… I don’t eat mountains of crisps and white bread sandwiches. I’m not that person. But in the evening, if I’m having an attack of loneliness and depression, then the binge eating disorder becomes very, very difficult to manage.” (P5, Female, 65, Divorced, Very Severe). *Sleep difficulties* “Obviously a lot depends on mood. A lot depends on sleep or lack of it thereof. So, you can feel ghastly through lack of sleep and it really, well that’s it for the day really. You can’t really come away from that. You just have to get through it. That’s the loneliness on that day.” (P28, Female, 75, Divorced, Very Severe).
Age-related losses.	*Network reducing over time* “Lack of everything. Loss of everything. Loneliness, to me, is because everything in my life has been eroded away from me, that’s things I did, things I was, the person I was. My friends, I’ve said to you the wheelchairs, they’re dead, they’ve got dementia. And everything with me has just gone and there’s just this empty, there’s nothing to share anything with.” (P42, Female, 91, Married, Moderate). *Physical intimacy* “And lack of physical closeness too. So, when I, when a cuddle is a very rare thing and you get about six hugs a year. So, I think a lot of its- I think a lot of it is felt in a physical kind of way. Or you know, the lack of the physical aspect sort of brings it home to you in a way.” (P39, Female, 70, Separating, Severe). *Emotional intimacy* “I guess loneliness by definition is just being alone in a room, but it can also be, being alone in a room when you’re with other people. Because you’re the one that doesn’t have anyone to go back to. Yes, so the shared experience is big for me.” (P32, Male, 70, widowed, moderate) “Sometimes I mean, I think my husband is the one that makes me feel the most alone, even when he’s here, because he doesn’t seem like he’s really here, you know he’s too, concentrating on something else.” (P7, Female, 65, Married, Very Severe) *Unable to do the same activities* “I couldn’t go and enjoy all the things you want to do. So that would make me feel alone because I can’t do them. And it annoys me that I can’t because I want to join in on the things. But as you become older and more unable to do things, you know, you are limited.” (P17, Female, 68, Single, Severe) *Pain* “And not knowing from one day to the next how bad the pain levels are going to be with osteoarthritis, that being indoors stops me or helps me from having the embarrassment of being outside and not being able to, if I fell, not being able to get up or not having anybody around to help.” (P29, Female, 71, Widowed, Severe)
Individual differences in overcoming loneliness.	*Keep busy* “Well I’d finished doing a task so I had time before I got motivated to do another one but I feel lonely then. But plus I get very tired at my sort of age. It’s easy to get tired so difficult to start something new when I’ve just finished something. So empty space there and then I get lonely then.” (P12, Male, 68, Single, Severe) *Motivation* “I think it’s a downward spiral. I once you feel lonely, you don’t want to do things because you’re not in a good place. I think that makes it worse, it’s not as if I’m sitting there and saying, I’m lonely I need to do something about this so that we can go out. It’s, I’m lonely I can’t do it.” (P1, Female, 68, Widowed, Moderate) *Bi-directional relationship with depression* “Well they’re pretty much inexplicably linked … it makes me feel isolated, empty and that does link to sort of a level of depression. I don’t know it’s hard to explain, as I say they’re so interlinked that if I’m honest if you’re feeling lonely and not connected then your mental health is going to suffer.” (P11, Male, 69, Single, Very Severe) *Cycle of loneliness* “You know, it’s frustration as well, because, of course, I can’t do things. I get frustrated, and then I get cross with myself because I’m feeling miserable because I can’t go and see anybody. But then I don’t make the effort to go and see anybody. So I’m partly to blame for my own loneliness.” (P7, Female, 65, Married, Very Severe)
